# Quantitative Structure Activity Relationship Models for the Antioxidant Activity of Polysaccharides

**DOI:** 10.1371/journal.pone.0163536

**Published:** 2016-09-29

**Authors:** Zhiming Li, Kaiying Nie, Zhaojing Wang, Dianhui Luo

**Affiliations:** Department of Bioengineering and Biotechnology, Huaqiao University, Fujian Xiamen, 361021, China; Bharathidasan University, INDIA

## Abstract

In this study, quantitative structure activity relationship (QSAR) models for the antioxidant activity of polysaccharides were developed with 50% effective concentration (EC_50_) as the dependent variable. To establish optimum QSAR models, multiple linear regressions (MLR), support vector machines (SVM) and artificial neural networks (ANN) were used, and 11 molecular descriptors were selected. The optimum QSAR model for predicting EC_50_ of DPPH-scavenging activity consisted of four major descriptors. MLR model gave EC_50_ = 0.033Ara-0.041GalA-0.03GlcA-0.025PC+0.484, and MLR fitted the training set with R = 0.807. ANN model gave the improvement of training set (R = 0.96, RMSE = 0.018) and test set (R = 0.933, RMSE = 0.055) which indicated that it was more accurately than SVM and MLR models for predicting the DPPH-scavenging activity of polysaccharides. 67 compounds were used for predicting EC_50_ of the hydroxyl radicals scavenging activity of polysaccharides. MLR model gave EC_50_ = 0.12PC+0.083Fuc+0.013Rha-0.02UA+0.372. A comparison of results from models indicated that ANN model (R = 0.944, RMSE = 0.119) was also the best one for predicting the hydroxyl radicals scavenging activity of polysaccharides. MLR and ANN models showed that Ara and GalA appeared critical in determining EC_50_ of DPPH-scavenging activity, and Fuc, Rha, uronic acid and protein content had a great effect on the hydroxyl radicals scavenging activity of polysaccharides. The antioxidant activity of polysaccharide usually was high in MW range of 4000–100000, and the antioxidant activity could be affected simultaneously by other polysaccharide properties, such as uronic acid and Ara.

## Introduction

In our normal metabolism process, oxygen free radicals and non-oxygen free radicals are continuously produced, and lower concentrations of free radical can play a crucial role in regular physiological functions [1–5]. However, many diseases, such as cardiovascular diseases, diabetes, aging and cancer, can be conducted by unregulated overproduction of free radicals [6–8]. Thus, it is essential to develop natural and effective antioxidants [9]. Previously reports revealed that many natural polysaccharides possess potent scavenging activities of free radicals and can be used as potential antioxidants [10–11]. It is always impossible to obtain a large quantity of experimental data because of a lack of perfect data sites, and so the study on relationship between bioactivities and the properties of polysaccharides by model forecast approach was relatively poor [12].

The quantitative structure-activity relationship (QSAR) model, which use relevant molecular physico-chemical properties to predict important treatment responses, is considered as an alternative to the experimental evaluation [13]. It has gained increasingly attention, and a variety of QSAR methods have been developed for water treatment process selection, membrane separation and adsorption etc [14–15].

To date, QSAR models for predicting the bioactivities of polysaccharides have seldom been developed. A study reported the relationship between monosaccharide composition ratio and macrophage stimulatory activity by model forecast approach [12]. To obtain theoretical supports for applications of polysaccharides from natural products, the main aim of this work was to establish reliable soft measurement models to predict performance and study the relationship between polysaccharide properties and antioxidant activities of polysaccharides by QSAR. In our QSAR studies, multiple linear regression (MLR) method, and the nonlinear methods including artificial neural network (ANN) and support vector machine (SVM) were used.

## Materials and Methods

### Data set

The present study showed that the antioxidant activity of polysaccharide has related with many factors, including monosaccharide composition [16], uronic acid (UA), molecular weight (MW), protein content (PC) and sulfate group content et al [17]. In the data selection, we chose natural purified polysaccharides without sulfate groups to study QSAR models for predicting antioxidant activities of polysaccharides. A various set of polysaccharides and their antioxidant activities were collected from different published papers [18–45]. Antioxidant activities of polysaccharides were represented by the 50% effective concentration (EC_50_). To set up a more reliable model, we selected 141 compounds. The detailed publication lists with corresponding antioxidant activities and compounds were given. The normalization process was adopted in the distribution of the parameters with 2 as the bottom of the log logarithm, and MW was divided by 10000 in the normalization process.

In models, a training data set was applied to develop the model. A test set, which was never included during their development, was used to validate the predictive power of model [46–47]. The training set and test set were chosen by random distribution.

### Descriptors

The structure of polysaccharide was complex and could be represented by variety of descriptors. However, the major composition of polysaccharide was monosaccharide joined together by glycosidic bonds, which was essential to their bioactivities, so we used monosaccharide composition as descriptors. The following descriptors of monosaccharide composition were considered for modeling EC_50_ values in MLR, ANN and SVM analysis. Descriptors of monosaccharide composition: rhamnose (Rha), arabinose (Ara), mannose (Man), glucose (Glc), galactose (Gal), fucose (Fuc), xylose (Xyl), ribose (Rib), glucuronic acid (GlcA) and galacturonic acid (GalA). Usually, gas chromatography (GC) and high-performance liquid chromatography (HPLC) were performed for the identification and quantification of monosaccharide composition. For HPLC analysis, glucuronic acid (GlcA) and galacturonic acid (GalA) could not be identified. Thus, total uronic acid (UA) could be determined by other methods, such as the sulfuric acid carbazole method, and then UA was also used as a descriptor in our models. The descriptors of PC and MW were also adopted in models. STATISTIC.10 method was used to establish SVM, MLP and ANN models, and the picture was drawn by using RStudio (Version 0.99.902–2009–2016 RStudio, Inc.).

### Linear model generation

There were primarily two different approaches for choosing a descriptor subset in MLR, and they were filter and wrapper methods. The procedure of filter method was that setting and filtering descriptors were supposed to generate the top priority subset before training. However, the learning algorithm was wrapped into the selection procedure in the wrapper method [48]. In MLR, we used wrapper as the target learning algorithm. The training data set was applied only for selecting descriptor. At first, we employed a two-dimensional research method. It was a combination of forward and backward search. Then we assessed the selected descriptors on the target learning algorithm. In the learning process, we used 10 fold cross validation method. In stepwise MLR analysis, we selected training descriptor sets and then established a linear model [49].

### Artificial neural network and Support vector machines

It was appropriate for artificial neural network (ANN) to model nonlinear relationship. We can find many reviews about ANN research and its application in QSAR studies [49–51]. In this study, we employed multi-layer perceptron (MLP) [52] and three layer reverse Back-Propagation (BP) network. In the back-propagation ANN, we utilized the technique of supervised learning, and the trained network was trained by minimizing the squared error of the network’s output. The first step of training model was to confirm the number of layers and neurons in each player. The second step was to optimize the learning rate as well as momentum parameters. In the input layer, the architecture of the network was composed of eleven neurons, which were the eleven relative descriptors chosen. In the output layer, there was one neuron, i.e. EC_50_ values of the antioxidant activity. In all the layers, logistic function was applied. In the hidden layer, through changing the number of neurons, we got the lowest RMSE and highest correlation coefficient. We applied 30% of the training data set for verification. The verification was employed to hinder from the over fitting. All of optimization process were taken with 10 fold cross validation [53].

Support vector machines (SVM) was originally developed for the classification problem, and SVM has been used to solve nonlinear regression estimation. Nowadays, SVM has demonstrated much success in QSAR and quantitative structure-property relationship (QSPR) studies [54–57]. We selected support vector machine classifier method (epsilon-SVM) which was most commonly used in QSPR and QSAR studies to optimize the value of kernel parameter g (gamma) [53].

### Validation techniques and model performance evaluation

We used a 10 fold cross validation technique. This procedure divided the data set into 10 folds or groups, created the model using 9 of the sets, and tested it on the remaining group. When the procedure was repeated, each of the 10 groups had served as a test group. The root mean square error (RMSE) was calculated, averaged, and then used to evaluate the predictive performance of three models.

## Results and Discussion

### Models for the DPPH scavenging activity of polysaccharides

**Fig 1:**
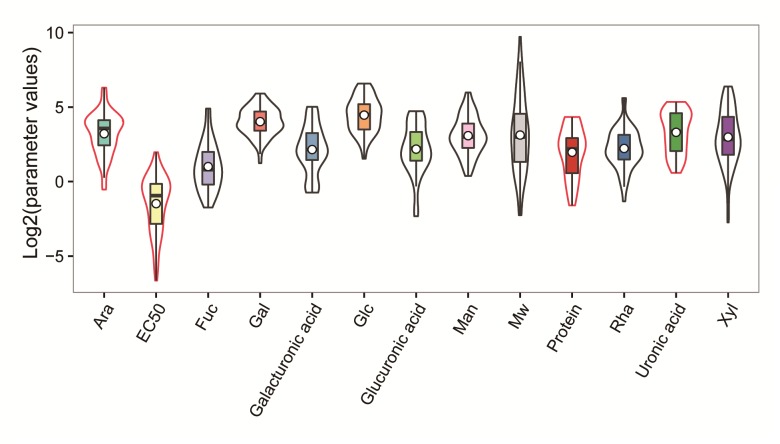
Data distribution of parameter.

The data was divided into two parts using random classification. One was the training set, the other was the test set. The entire data set including 74 compounds was divided into two clusters. The test set of 22 compounds was chosen randomly from this cluster, and the remaining compounds were used as the train set. Compound number 4, 5, 7, 9, 17, 20, 25, 30, 31, 32, 33, 34, 36, 38, 44, 54, 57, 62, 63, 64, 69,73 were selected as the test set, and the rest of the compounds were the train set. The test set and train set were given in Table 1. The data distribution of parameter was shown in Fig 1, the data distribution was uniform, and no other single variable values was close to EC_50_ values distribution (-6, 2). The shape of data distribution from EC_50_ and Ara was similar, which indicated that there was a certain relation between them. In addition to MW, other physical quantities were all the components of polysaccharides, so MW was used to establish the model by itself.

**Table 1:** Polysaccharides data set with descriptors and EC_50_ values of the DPPH scavenging activity.

No	Name^a^	Rha^b^	Ara^c^	Man^d^	Glc^e^	Gal^f^	Fuc^g^	Xyl^h^	GlcA^i^	GalA^j^	UA^k^	PC^l^	EC_50_	Refs
**1**	S1 (Glu)	0	0	22.13	28.33	13.89	0	31.48	4.03	0	4.03	0	1.104	18
**2**	S1 (Visco)	0	0	63.01	10.37	16.06	0	4.7	5.87	0	5.87	0	0.794	18
**3**	GBP50S2	46.7	0	42.2	0	11.1	0	0	0	0	0	0	0.412	19
**4**	LBP-s80	0	62.9	13.2	2.9	12.5	3.8	4.7	0	0	17.07	0.69	2.97	20
**5**	LBP-s75	0	56.8	19.2	3.6	10.2	4.9	5.3	0	0	35.69	1.3	1.98	20
**6**	WB1	0	0	12.5	59.4	9.76	11.7	6.64	0	0	0	0	0.12	21
**7**	WB2	0	0	7.7	26	24.7	28.4	13.2	0	0	0	0	0.31	21
**8**	WB3	0	0	9.7	18.6	24.5	29.9	17.3	0	0	0	0	0.21	21
**9**	IOP40	4.4	4.2	9.8	40	14.5	3.3	9.6	9.7	4.6	5	2.4	0.88	22
**10**	IOP60	9.7	5.6	8	32.2	12.6	0	22.9	4.7	4.4	2.2	3.2	0.697	22
**11**	IOP80	11	5.6	9.7	31.3	8.5	0	25.3	2.8	3.9	1.5	4.6	1.19	22
**12**	FUP-1	0	0	0	9.81	6.78	0	83.41	0	0	0	0	0.47	23
**13**	CLP-2	3.3	2.1	14.5	48	28	0	4.1	0	0	23.59	1.48	0.86	24
**14**	CLP-3	0	0	8.6	56	29.4	0	6	0	0	17.06	0.95	1.27	24
**15**	TYAP-1	0	78.98	0	5.74	10.6	0	4.68	0	0	0	0	3.92	25
**16**	PV-P1	0	24.2	1.9	8.3	9.7	0	55.9	0.8	3.5	3.4	1.22	0.878	26
**17**	PV-P2	3.6	15.7	14.4	16	21.6	0	28.7	0.3	5.4	5.7	4.22	0.169	26
**18**	PV-P3	6.1	16.5	16.1	11.2	13.3	0	36.8	0.2	7.9	8.1	7.09	0.048	26
**19**	Control-EPS	6.1	14.6	20.4	20.7	24.2	0	14	0	0	0	19.75	2.3	27
**20**	Control-IPS1	3.1	7.2	28	36.7	19	0	6	0	0	0	17.57	1.08	27
**21**	Tween 80-IPS1	3.3	2.4	6.9	73.4	11.4	0	2.6	0	0	0	16.72	0.74	27
**22**	Tween 80-IPS2	1.4	5.2	18.3	60.9	12.1	0	2.1	0	0	0	15.61	0.84	27
**23**	CPSI	0	0	27	73	0	0	0	0	0	0	0	0.23	28
**24**	G1	10.9	1.2	6.2	52.5	14.9	0	14.3	0	0	4.2	6.49	0.34	29
**25**	G2	12.2	0.8	4.9	56	16.2	0	9.9	0	0	7.45	5.11	0.56	29
**26**	G3	12.2	3.8	3.2	50.2	12.5	0	18.1	0	0	1.92	3.62	0.87	29
**27**	P1	11.4	30.3	1.5	9.2	44.4	0	3.2	0	0	0	0	0.62	30
**28**	P2	10.4	22.1	3.1	11.2	53.1	0	0	0	0	0	0	1.07	30
**29**	CP	1.2	15.6	7.5	28.2	24.7	0	5.4	4.8	12.6	17.4	7.57	0.09	31
**30**	SCG	0	19.93	4.43	15.37	60.27	0	0	0	0	0	0	0.7	32
**31**	PNMP2	0	5.78	28.62	14.42	41.57	7.24	2.37	0	0	0	0	0.3297	16
**32**	PNMP3	0	3.45	26.58	21.55	36.42	8.44	3.56	0	0	0	0	0.1516	16
**33**	GLP60	0	0	3.2	85.9	8.2	1.5	0	0	0	0	0	0.97	8
**34**	GLP80	0	0	9.4	79.4	5.4	1.1	0	0	0	0	0	0.72	8
**35**	GLP	0	0	4.8	86.5	6.1	1.2	0	0	0	0	0	0.9	8
**36**	LLPs-D	6.83	2.73	9.2	19.23	58.19	0.57	3.25	0	0	0	0	0.38	33
**37**	LLPs-L	5.03	19.39	6.07	22.82	37.45	7.04	2.21	0	0	0	0	0.99	33
**38**	SMWP-1	0	0	27	34	11	0	28	0	0	0	0.53	0.13	34
**39**	EAP40-1	2.63	0	36	46.79	14.58	0	0	0	0	0	0.33	0.28	35
**40**	EAP60-1	3.37	2.28	2.89	43.61	37.67	0	10.18	0	0	0	0.48	0.52	35
**41**	CMP-1	4.2	0	0	95.8	0	0	0	0	0	0	0	1.15	36
**42**	GPA1	0.4	21.2	10.6	13.8	27.5	2.2	0	14.8	9.5	23.04	3.75	0.08	37
**43**	GPA2	0.8	15.6	8.2	18	21.4	1.6	1.6	18	14.8	32.79	4.38	0.06	37
**44**	GPA3	3.8	7.5	6.3	34.3	16.3	1.3	3.1	24.3	3.1	27.01	5.53	0.03	37
**45**	Ac-CP1	2.5	16.4	5.6	17.6	27.5	0	1.8	2.6	26	15.78	7.25	0.06448	38
**46**	Ac-CP2	2.8	15.3	6	14.3	26.2	0	2	2.9	30.5	25.99	6.93	0.07829	38
**47**	Ac-CP3	2.2	15.6	5.6	9.8	29.9	0	1.1	3.4	32.4	27.43	7.09	0.07804	38
**48**	CP	1.2	15.6	7.5	28.1	24.8	0	5.4	4.8	12.6	16.14	7.57	0.09837	38
**49**	WFPs	5.2	18.5	3.5	15.9	21.3	0	7.4	3.3	24.9	28.1	0	0.007	39
**50**	APs-2-1	4.6	8	0	32.3	24.2	0	21.1	0	9.8	9.8	1.9	0.4545	40
**51**	APs-3-1	1.5	2.8	0	35.1	34	0	16.7	2	7.9	7.9	1.3	0.2243	40
**52**	PTPS-3	6.82	26.22	13.83	10.23	39.34	3.21	0.35	0	0	40.66	13.27	1.72	41
**53**	PTPS-5	15.98	20.84	15.29	6.08	40.33	1.68	0.15	0	0	40.44	19.96	1.45	41
**54**	PSS-EPS	8.2	7.7	24	35.3	15.4	0	9.4	0	0	0	20.19	1.497	42
**55**	UKLOxa	5.5	10.2	6.1	11.3	28.4	0.3	7.2	26.5	4.5	31	0	0.0546	43
**56**	UKLK1	3.6	6.1	5.8	10.9	9.4	1.8	5.03	6.5	2.9	9.4	0	0.136	43
**57**	UKLK4	2.5	6.6	2.6	8.3	6.6	1.1	64.4	7.1	0.8	7.9	0	0.6023	43
**58**	UKSOxa	5.6	9.9	11.8	18.7	16.9	0.4	9.4	24.3	3	27.3	0	0.0165	43
**59**	UKSOxa-PG	8.6	15.7	7.1	12.4	26.1	0.5	15.4	10.2	4	14.2	0	0.3751	43
**60**	UKSK1	3.5	4	1.9	6.2	3.6	0.7	76.5	2.8	0.8	2.6	0	0.177	43
**61**	UKSK4	3.6	8.7	3.7	8.3	6.2	0.9	66	1.9	0.7	2.6	0	0.0038	43
**62**	PMBOxa	8.8	11.9	9.5	23.8	18.3	0.7	8.4	16.3	2.3	18.6	0	0.0217	43
**63**	PMBOxa-PG	6.7	12.4	10.3	26.6	25.5	0.8	6.7	8	3	11	0	0.144	43
**64**	PMBK1	4.8	13.3	4.8	17.2	13.7	1.5	37.5	5.3	1.9	7.2	0	0.3143	43
**65**	PMBK4	2.4	18.4	2.3	9.9	8.9	2.6	51.9	3	0.6	3.6	0	0.6547	43
**66**	AMBOxa	10.9	14.5	4.2	25.9	14.9	0.5	6.1	16.8	6.2	23	0	0.0184	43
**67**	AMBOxa-PG	17.4	22.6	4	5.9	16.6	0.6	7.1	20.8	5	25.8	0	0.3533	43
**68**	AMBK1	2	4.6	1.9	32.2	10.7	1.8	44.2	1.5	1.1	2.6	0	0.1093	43
**69**	AMBK4	3.2	27.2	1.3	17.1	6.5	2.1	40.6	1.3	0.7	2	0	1.4203	43
**70**	PS1	0.79	0.69	60.51	32.66	2.35	2.98	0	0	0	0	0	1.21	44
**71**	PS2	10.96	5.81	36.16	26.92	14.55	4.52	1.04	0	0	0	0	0.73	44
**72**	PS3	48.55	10.73	7.35	11.41	13.85	4.62	3.45	0	0	0	0	0.67	44
**73**	WKCP-N	0	2.22	0	91.95	5.83	0	0	0	0	0	0	0.61	45
**74**	WKHP-N	0	12.9	0	73.71	10	0	1.34	2.45	0	3.2	0	1.08	45

^a^name from reference

^b^rhamnose

^c^arabinose

^d^mannose

^e^glucose

^f^ galactose

^g^fucose

^h^xylose

^i^glucuronic acid

^j^galacturonic acid

^k^uronic acid

^l^protein content

### MLR results

**Fig 2:**
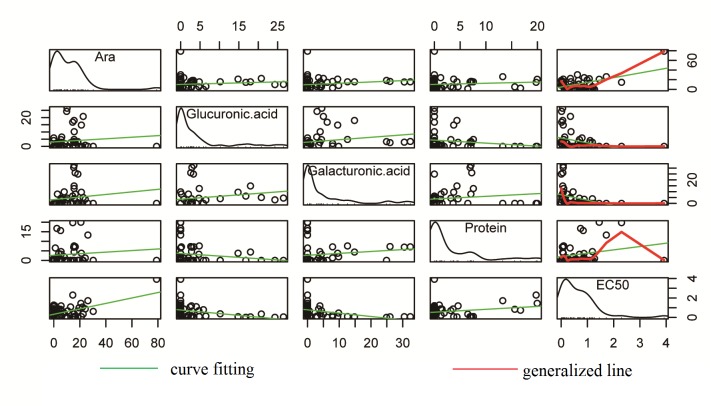
Correlation between the matrix of parameters and EC_50_ value of the DPPH scavenging activity.

**Fig 3:**
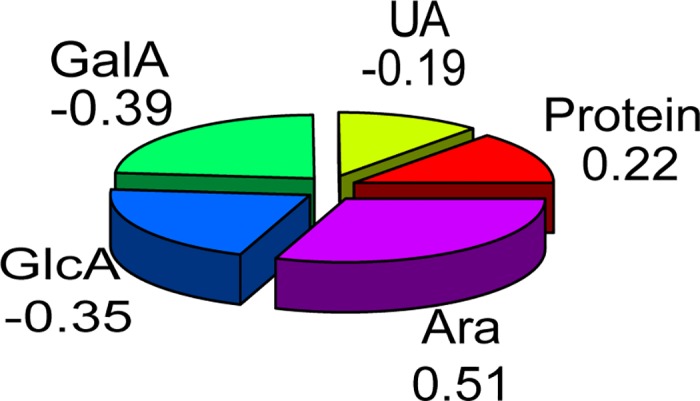
Proportion of the parameters effecting on EC_50_ value of the DPPH scavenging activity.

In this study, the training data set of 52 compounds was used. A stepwise linear regression analysis was used to determine the relationship between the dependent variable of EC_50_ and the independent variables of uronic acid (UA), protein content (PC) and monosaccharide compositions (Rha, Ara, Man, Glc, Gal, Fuc, Xyl, GlcA and GalA). To achieve this goal, regression analysis was implemented by using the forward stepwise. In stepwise regression procedures, the first was to choose the most correlated independent variable, and then to select independent variable which was most correlated with the remaining variance in the dependent variable. This procedure was to increase the additional independent variable with R-squared (R^2^) which was not changing until a significance of at least 80%. Accordingly, the variables of Ara, GalA, GlcA and PC were included in the regression model. The relationship between the matrix of parameters and EC_50_ was shown in Fig 2. One variable data was used as the abscissa, another variable data was used as ordinate, and all points had been portrayed by the matrix scatter plot. From the diagonal we can see that the distribution of the data was all similar in shape. Fig 3 showed the correlation between model parameters and EC_50_, and the proportion of Ara, GalA and GlcA accounted 0.51, 0.39 and 0.35, respectively, which indicated that they had the most effect on EC_50_. In Fig 3, we can see that EC_50_ had a positive correlation with Ara and PC, and it has negative correlation with GalA and GlcA, which was consistent with the model given in equation. The regression Eq 1, which could be obtained through the statistical analysis, was as follows. Because the effect of UA on EC_50_ was little, UA was not added to the model equation. The linear model selected four major relevant descriptors, and gave a stable model with R = 0.807 and RMSE = 0.423.

$$\begin{array}{l} {\mathrm{EC}}_{50}=0.033\mathrm{Ara}-0.041\mathrm{GalA}-0.03\mathrm{GlcA}-0.025\mathrm{PC}+0.484\\ R=0.807\;\mathrm{SE}=0.423\;F=21.979\;\text{p}=2.82\;{\text{E}}^{-10}<0.001 \end{array}$$ (1)

**Fig 4:**
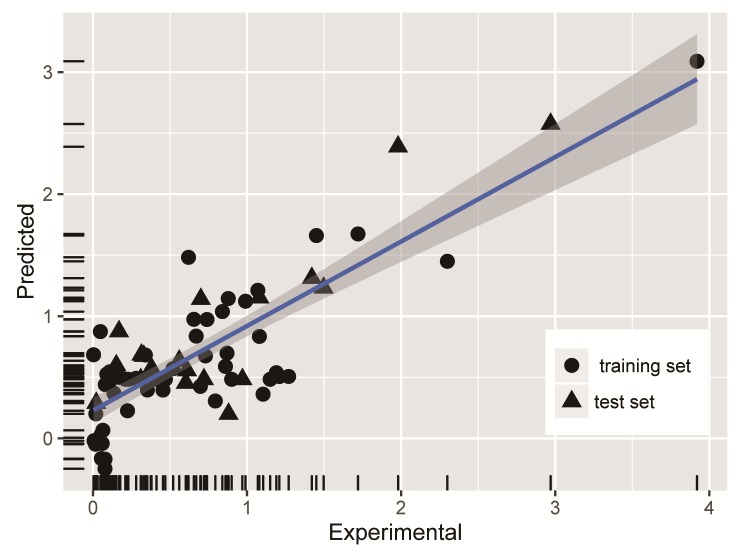
A comparison of experimental vs predicted EC_50_ using MLR method.

In the model, R value was 0.807 (p <0.001), fit indicators of the model were acceptable, the model was coincided with the data structure, and Ara, GalA, GlcA, PC and EC_50_ were significant correlation. The predicted EC_50_ values of the training and test set by using the MLR equation were given in the Table 2. Predicted values and experimental values of EC_50_ in two sets of data were plotted and shown in Fig 4. Most of the data were distributed from 0 to 1.5, and there were some predicted and negative values existing in the left lower corner. The experimental values of these negative values were between 0 and 0.2, which could be accepted. Experimental values and predicted points were distributed in two sides of the curve fitting, and most point of test set distributed among the prediction set, which illustrated that the establishment of training set used for the multiple regression model was very good to predict the numerical value of test set. The above linear model was applied to predict the 22 test data set, and these test data were never used in model building. The result showed R = 0.872, RMSE = 0.361 and p = 1.245E^-7^, which showed that there was a significant correlation. Multiple linear regressions (MLR) established the relationship between the dependent variable of EC_50_ and the independent variable of polysaccharide properties. The results showed that the statistics for MLR equation were good, and it also offered some views about the polysaccharide properties influences on DPPH-scavenging activity of polysaccharides.

**Table 2:** Experimental and predicted values of EC_50_ for the DPPH-scavenging activity of polysaccharides using MLR, ANN and SVM models.

No	Name^a^	Exp	MLR		SVM		ANN	
			Predict	Residue	Predict	Residue	Predict	residue
**1**	S1 (Glu)	1.104	0.361582	0.742418	0.366201	0.737799	0.953944	0.150056
**2**	S1 (Visco)	0.794	0.305859	0.488141	0.706948	0.087052	0.956870	-0.162870
**3**	GBP50S2	0.412	0.483627	-0.071627	0.608142	-0.196142	0.376554	0.035446
**4**	LBP-s80	2.97	2.575070	0.394930	1.958210	1.011790	3.430658	-0.460658
**5**	LBP-s75	1.98	2.388860	-0.408860	1.868621	0.111379	2.222439	-0.242439
**6**	WB1	0.12	0.483627	-0.363627	0.367456	-0.247456	0.395514	-0.275514
**7**	WB2	0.31	0.483630	-0.173630	0.040901	0.269099	0.181648	0.128352
**8**	WB3	0.21	0.483627	-0.273627	0.013756	0.196244	0.138245	0.071755
**9**	IOP40	0.88	0.200140	0.679860	0.123591	0.756409	0.508640	0.371360
**10**	IOP60	0.697	0.425514	0.271486	0.291228	0.405772	0.814391	-0.117391
**11**	IOP80	1.19	0.537777	0.652223	0.378394	0.811606	0.955650	0.234350
**12**	FUP-1	0.47	0.483627	-0.013627	0.274257	0.195743	0.145075	0.324925
**13**	CLP-2	0.86	0.589228	0.270772	0.723493	0.136507	0.863565	-0.003565
**14**	CLP-3	1.27	0.506954	0.763046	0.649755	0.620245	1.104942	0.165058
**15**	TYAP-1	3.92	3.088460	0.831540	2.438504	1.481496	3.770749	0.149251
**16**	PV-P1	0.878	1.145072	-0.267072	0.681146	0.196854	0.592526	0.285474
**17**	PV-P2	0.169	0.876230	-0.707230	0.577149	-0.408149	0.551492	-0.382492
**18**	PV-P3	0.048	0.874380	-0.826380	0.516508	-0.468508	0.321103	-0.273103
**19**	Control-EPS	2.3	1.450109	0.849891	1.258621	1.041379	2.311131	-0.011131
**20**	Control-IPS1	1.08	1.152520	-0.072520	1.093392	-0.013392	1.781373	-0.701373
**21**	Tween 80-IPS1	0.74	0.973340	-0.233340	0.935877	-0.195877	0.419414	0.320586
**22**	Tween 80-IPS2	0.84	1.038431	-0.198431	0.979781	-0.139781	0.776261	0.063739
**23**	CPSI	0.23	0.483627	-0.253627	0.747002	-0.517002	0.339270	-0.109270
**24**	G1	0.34	0.682566	-0.342566	0.618879	-0.278879	0.678410	-0.338410
**25**	G2	0.56	0.635490	-0.075490	0.619038	-0.059038	0.912242	-0.352242
**26**	G3	0.87	0.697843	0.172157	0.614961	0.255039	0.920268	-0.050268
**27**	P1	0.62	1.482949	-0.862949	1.139900	-0.519900	0.912532	-0.292532
**28**	P2	1.07	1.212505	-0.142505	0.991519	0.078481	0.774189	0.295811
**29**	CP	0.09	0.525923	-0.435923	0.348891	-0.258891	0.112901	-0.022901
**30**	SCG	0.7	1.140940	-0.440940	1.009837	-0.309837	0.745078	-0.045078
**31**	PNMP2	0.3297	0.674260	-0.344560	0.595812	-0.266112	0.513151	-0.183451
**32**	PNMP3	0.1516	0.597410	-0.445810	0.504217	-0.352617	0.423722	-0.272122
**33**	GLP60	0.97	0.483630	0.486370	0.704868	0.265132	0.650600	0.319400
**34**	GLP80	0.72	0.483630	0.236370	0.697402	0.022598	0.519819	0.200181
**35**	GLP	0.9	0.483627	0.416373	0.718195	0.181805	0.627824	0.272176
**36**	LLPs-D	0.38	0.573660	-0.193660	0.623545	-0.243545	0.573738	-0.193738
**37**	LLPs-L	0.99	1.123127	-0.133127	0.794887	0.195113	0.608499	0.381501
**38**	SMWP-1	0.13	0.496640	-0.366640	0.528723	-0.398723	0.605999	-0.475999
**39**	EAP40-1	0.28	0.491730	-0.211730	0.688481	-0.408481	0.330165	-0.050165
**40**	EAP60-1	0.52	0.570610	-0.050610	0.593314	-0.073314	0.667831	-0.147831
**41**	CMP-1	1.15	0.483627	0.666373	0.800434	0.349566	0.948406	0.201594
**42**	GPA1	0.08	0.440122	-0.360122	0.275073	-0.195073	0.109224	-0.029224
**43**	GPA2	0.06	-0.041681	0.101681	-0.019261	0.079261	0.043427	0.016573
**44**	GPA3	0.03	0.004720	0.025280	0.116442	-0.086442	0.087376	-0.057376
**45**	Ac-CP1	0.06448	0.065797	-0.001317	-0.003399	0.067879	0.022510	0.041970
**46**	Ac-CP2	0.07829	-0.170541	0.248831	-0.094824	0.173114	0.017536	0.060754
**47**	Ac-CP3	0.07804	-0.249175	0.327215	-0.117474	0.195514	0.017260	0.060780
**48**	CP	0.09837	0.525923	-0.427553	0.343823	-0.245453	0.125297	-0.026927
**49**	WFPs	0.007	-0.019404	0.026404	0.007187	-0.000187	0.016533	-0.009533
**50**	APs-2-1	0.4545	0.395343	0.059157	0.257761	0.196739	0.074447	0.380053
**51**	APs-3-1	0.2243	0.225857	-0.001557	0.200810	0.023490	0.147594	0.076706
**52**	PTPS-3	1.72	1.674231	0.045769	1.524571	0.195429	1.532898	0.187102
**53**	PTPS-5	1.45	1.661066	-0.211066	1.636562	-0.186562	1.546828	-0.096828
**54**	PSS-EPS	1.497	1.233340	0.263660	1.142511	0.354489	1.906331	-0.409331
**55**	UKLOxa	0.0546	-0.165611	0.220211	0.036641	0.017959	0.072630	-0.018030
**56**	UKLK1	0.136	0.369956	-0.233956	0.332098	-0.196098	0.164552	-0.028552
**57**	UKLK4	0.6023	0.453730	0.148570	0.185824	0.416476	0.087298	0.515002
**58**	UKSOxa	0.0165	-0.047842	0.064342	0.094666	-0.078166	0.070345	-0.053845
**59**	UKSOxa-PG	0.3751	0.529760	-0.154660	0.335927	0.039173	0.159281	0.215819
**60**	UKSK1	0.177	0.498201	-0.321201	0.232651	-0.055651	0.102440	0.074560
**61**	UKSK4	0.0038	0.684537	-0.680737	0.368651	-0.364851	0.231762	-0.227962
**62**	PMBOxa	0.0217	0.288880	-0.267180	0.250147	-0.228447	0.105432	-0.083732
**63**	PMBOxa-PG	0.144	0.528240	-0.384240	0.417461	-0.273461	0.559193	-0.415193
**64**	PMBK1	0.3143	0.684450	-0.370150	0.393583	-0.079283	0.306324	0.007976
**65**	PMBK4	0.6547	0.975208	-0.320508	0.549260	0.105440	0.626754	0.027946
**66**	AMBOxa	0.0184	0.200785	-0.182385	0.172081	-0.153681	0.053022	-0.034622
**67**	AMBOxa-PG	0.3533	0.395625	-0.042325	0.279658	0.073642	0.083612	0.269688
**68**	AMBK1	0.1093	0.545151	-0.435851	0.353599	-0.244299	0.546221	-0.436921
**69**	AMBK4	1.4203	1.312850	0.107450	0.859646	0.560654	1.691224	-0.270924
**70**	PS1	1.21	0.506384	0.703616	0.824544	0.385456	1.219299	-0.009299
**71**	PS2	0.73	0.675246	0.054754	0.649997	0.080003	0.467911	0.262089
**72**	PS3	0.67	0.837512	-0.167512	0.612208	0.057792	0.702244	-0.032244
**73**	WKCP-N	0.61	0.556840	0.053160	0.816849	-0.206849	0.776698	-0.166698
**74**	WKHP-N	1.08	0.834885	0.245115	0.884773	0.195227	1.335107	-0.255107

^a^name from reference

### ANN results

**Fig 5:**
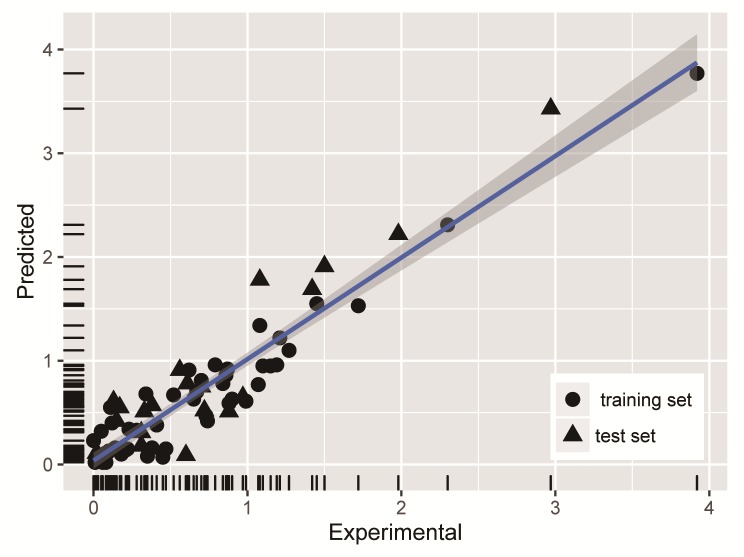
A comparison of experimental vs predicted EC_50_ using ANN method.

Polysaccharide properties were considered as the input layer node in neural networks, and EC_50_ values of the DPPH-scavenging activity was the output layer node. Numbers of nodes had a great influence on the test results. The optimization was done with 10 fold cross validation, and 30% of test data were used for validation. Selected parameters of the number of neurons in the hidden layer were optimized by changing from 4 to 14, and it was worthy to mention that the initial value of 7 selected was optimal. The selected network adopted Broyden Fletcher Goldfard Shanno (BFGS) algorithm which was still seen as the best Quasi-Newton algorithm. When the entire training data was trained in the network with the optimized parameters, it gave R = 0.96 and RMSE = 0.018. The experimental and predicted values of EC_50_ for the train data using the ANN model were plotted and shown in Fig 5. The experimental value was abscissa, the point distribution of the prediction value for the y-coordinate was on both sides of the curve fitting from 0 to 1.5, and the point distribution was uniform and closed to each other. According to the view of point, the density of horizontal and vertical coordinates and the fitting effect were perfect. The predicted values of EC_50_ for the train and test data were given in the Table 2. The test set was used for prediction and gave R = 0.933 and RMSE = 0.055.

### SVM results

**Fig 6:**
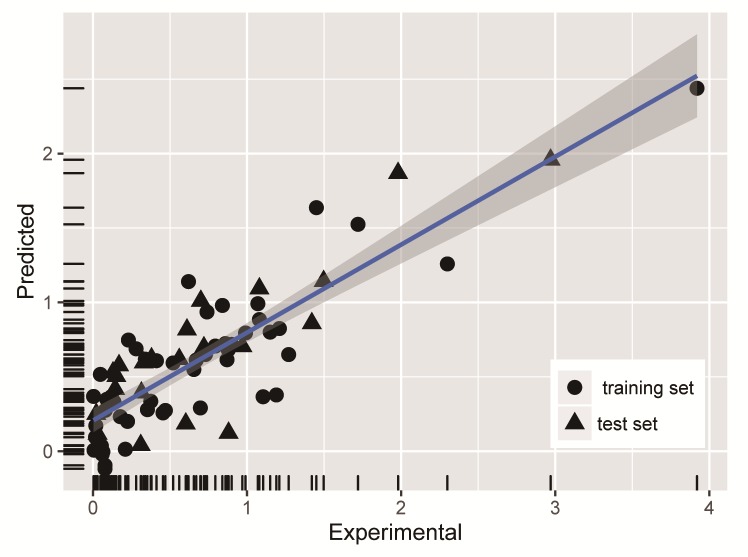
A comparison of experimental vs predicted EC_50_ using SVM method.

We selected radial basis function (RBF) kernel for function modeling in SVM, the best parameter C, g and ε were selected by using 10 fold cross validation, a SVM model was obtained by training the whole training set, and then the model was used for the test set. By varying the parameter values in the training set systematically, we optimized SVM parameters, and calculated RMSE of the model. The parameter value which gave the lowest RMSE was selected. The regularization parameter C controlled the alternate use between maximizing the margin and minimizing the training error. If the value of C was too small, then there was not sufficient stress on fitting the training data. To have a stable learning procedure, a large value of C should be set up first [57]. To discover an optimal value of C, the RMSE of SVM model with different C values was calculated. Then, this value C = 9 was selected as the optimal value. We achieved the selected parameters (g = 0.091, ε = 0.1, C = 9) and the final training running in the whole training set, and EC_50_ of the DPPH-scavenging activity was predicted. The predicted EC_50_ on the basis of this model was plotted and shown in Fig 6 and Table 2. The statistical parameters of this model were R = 0.851 and RMSE = 0.151 for the training set, and the test set was used for prediction and gave R = 0.865 and RMSE = 0.144.

### Comparison of MLR, ANN and SVM models

The statistical parameters obtained from the investigative models for train and test set were shown in Table 3. The error estimates were applied to model performance evaluation, and RMSE were lower for nonlinear models (SVM, ANN) generated by the machine learning methods than that by multiple linear regression. The correlation coefficients (R) given by SVM and ANN models were also higher than that by multiple linear regression. The above results indicated that the performances of nonlinear models SVM and ANN were better than that of a linear MLR model for the prediction of DPPH-scavenging activity of polysaccharides. The comparison of the nonlinear models demonstrated that ANN model accurately predicted the relationship between polysaccharide properties and the DPPH-scavenging activity for the train data set, and this was obviously evident from a lower RMSE (0.018) and a higher R (0.96) value. While ANN model was also the best one in the prediction of the test set.

**Table 3:** Comparison of MLR, ANN and SVM models for the DPPH scavenging activity of polysaccharides.

Method	Parameters	Training set	Test set
**MLR**	R	0.807	0.872
	RMSE	0.423	0.361
**ANN**	R	0.96	0.933
	RMSE	0.018	0.055
**SVM**	R	0.851	0.865
	RMSE	0.151	0.144

### Effect of MW on the scavenging activity of DPPH radical

Molecular weight was seen as an important indicator of the antioxidant activity of polysaccharides [20], so a single study was used to evaluate the relationship of MW and antioxidant activity of polysaccharides. Due to the relatively large difference in MW of polysaccharide from 2250 to 538500 (Table 4), MW was normalized before the analysis, the size of MW was taken with a base-8 of log, and the data was shown in Table 4 [58–66].

**Table 4:** MW and EC_50_ values of the DPPH scavenging activity.

Name^a^	EC_50_	Mw	Refs	Name	EC_50_	Mw	Refs	Name	EC_50_	Mw	Refs
PS	6.20	225000	58	CLP-3	1.27	60143	24	CMP-1	1.15	4300	36
PSPO-1a	1.43	18000	59	TYAP-1	3.92	115000	25	PPM	1.80	22000	64
LBP-80	5.33	70600	20	TYAP-2	4.11	479000	25	PPE	3.06	38000	64
LBP-s75	1.98	71700	20	TYAP-3	2.64	403000	25	GPA1	0.08	19600	37
LBP-s50	4.96	538500	20	PS1-1	6.81	67400	62	GPA2	0.06	10600	37
BSFP-1	7.40	13300	60	PS1-2	4.56	15400	62	GPA3	0.03	6700	37
WB2	0.31	28000	21	PS2-1	2.53	12100	62	AAP-2A	0.15	2252	65
WB3	0.21	19000	21	PNMP1	0.72	28400	16	RNLP I	0.20	14900	66
SP1	3.20	9192	61	PNMP2	0.33	31500	16	WKCP-N	0.61	9600	45
FUP-1	0.47	41000	23	PNMP3	0.15	26100	16	WKHP-N	1.08	113400	45
CLP-1	1.69	78754	24	AAP	3.29	27700	63	WKHP-A	3.34	169600	45
CLP-2	0.86	51257	24	EAP80-2	1.32	65313	35				

^a^name from reference

**Fig 7:**
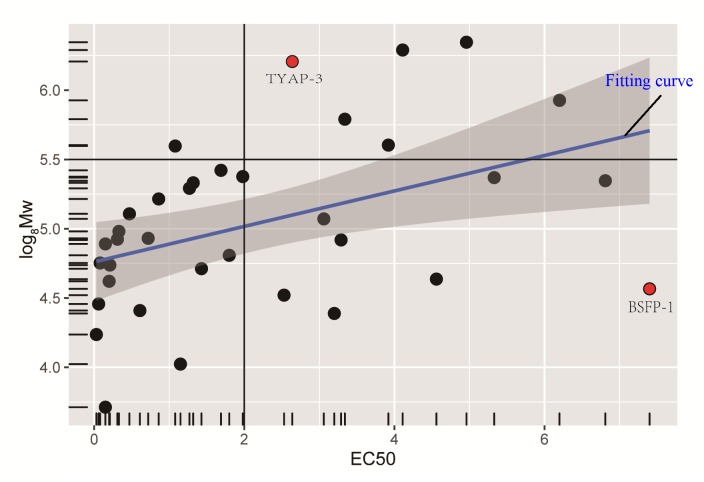
Correlation scatter plots of EC_50_ and MW.

We used EC_50_ values as the horizontal coordinate and established the correlation between EC_50_ and MW. As shown in Fig 7, the value of EC_50_ decreased with the decrease of MW, which indicated that the smaller MW could have the stronger DPPH free radical scavenging activity. This result was in accord with those reported in the literature [20, 59]. In Fig 7, it could also be found that there were some points which did not conform to the rules, such as TYAP-3 and BSFP-1. BSFP-1 had the smaller MW and a relatively larger EC_50_ value [60], which may be because BSFP-1 had no UA. TYAP-3 had larger MW, but its EC_50_ value was smaller. The reason may be that the content of Ara accounted for 45.82% in TYAP-3 [25]. Fig 7 showed that when the value of EC_50_ arranged from 0 to 2, the value of Y axis was 0–5.5, which indicated that MW was between 4000 and 100000.

According to the above results, we could conclude that the antioxidant activity of polysaccharide usually was higher in MW range of 4000–100000. However, MW was not the only factor, and the antioxidant activity could be affected by other polysaccharide properties, such as UA and Ara.

### Models for the hydroxyl radicals scavenging activity of polysaccharides

To make relationship models of monosaccharide composition and the hydroxyl radicals scavenging activity, the entire data set including 67 compounds was divided into two clusters [67–82]. The test set and the train set were given in Table 5.

**Table 5:** Polysaccharides data set with descriptors and their EC_50_ values of the hydroxyl radicals scavenging activity.

No	Name^a^	Rha^b^	Ara^c^	Man^d^	Glc^e^	Gal^f^	Fuc^g^	Xyl^h^	GlcA^i^	GalA^j^	UA^k^	PC^l^	EC_50_	Refs
**1**	PS-SI	0	27.3	18.2	9.1	45.4	0	0	0	0	0	0.5	0.21	67
**2**	CBP-1	0	0	35.9	12.8	51.3	0	0	0	0	0	0	0.638	68
**3**	GBP50S2	46.7	0	42.2	0	11.1	0	0	0	0	0	0	0.482	19
**4**	pMTPS-3	0	17.3	41.6	28.3	12.6	0	0	0	0	0	0	1.9	69
**5**	USEP40-1	7.95	8.42	37.34	17.94	28.36	0	0	0	0	0	0	0.376	70
**6**	USEP70-1	14.47	7.78	37.27	21.85	18.64	0	0	0	0	0	0	0.524	70
**7**	IOP40	4.4	4.2	9.8	40	14.5	3.3	9.6	9.7	4.6	5	2.4	0.58	22
**8**	IOP60	9.7	5.6	8	32.2	12.6	0	22.9	4.7	4.4	2.2	3.2	0.46	22
**9**	CLP-1	0	3.6	7.9	60.2	26.4	0	1.9	0	0	15.84	1.43	3.68	24
**10**	CLP-2	3.3	2.1	14.5	48	28	0	4.1	0	0	23.59	1.48	1.29	24
**11**	CLP-3	0	0	8.6	56	29.4	0	6	0	0	17.06	0.95	2.8	24
**12**	GPS-2	44.7	20.9	0	3.6	10.8	0	19.9	0	0	0	0	0.069	71
**13**	P70-1	0	0	56	18	26	0	0	0	0	0	0	0.548	65
**14**	PS1-1	0	0	89.5	7.3	3.2	0	0	0	0	0	1.67	1.14	62
**15**	PS1-2	0	0	71.1	3.7	25.2	0	0	0	0	0	1.86	0.48	62
**16**	PS2-1	0	0	52.7	28	16.9	0	0	0	2.4	0	3.85	0.36	62
**17**	O.ficus-indica -p	15.3	45.5	0	39.2	0	0	0	0	0	0	0	0.6318	72
**18**	G1	10.9	1.2	6.2	52.5	14.9	0	14.3	0	0	4.2	6.49	1.88	29
**19**	G2	12.2	0.8	4.9	56	16.2	0	9.9	0	0	7.45	5.11	1.41	29
**20**	P1	11.4	30.3	1.5	9.2	44.4	0	3.2	0	0	0	0	2.38	30
**21**	P2	10.4	22.1	3.1	11.2	53.1	0	0	0	0	0	0	0.98	30
**22**	CP	1.2	15.6	7.5	28.2	24.7	0	5.4	4.8	12.6	0	7.57	0.37	31
**23**	SSP II-a	8.94	38.74	0	2.18	31.47	0	0	2.33	16.34	0	0	0.7782	73
**24**	PNMP2	0	5.78	28.62	14.42	41.57	7.24	2.37	0	0	0	0	0.7117	74
**25**	PNMP3	0	3.45	26.58	21.55	36.42	8.44	3.56	0	0	0	0	0.4336	74
**26**	LLPs-D	6.83	2.73	9.2	19.23	58.19	0.57	3.25	0	0	0	0	0.61	33
**27**	LLPs-L	5.03	19.39	6.07	22.82	37.45	7.04	2.21	0	0	0	0	0.92	33
**28**	SMWP-1	0	0	27	34	11	0	28	0	0	0	0.53	1.08	34
**29**	GRMP1	0	0	0	31.5	0	0	68.5	0	0	0	0	0.1472	16
**30**	EAP40-1	2.63	0	36	46.79	14.58	0	0	0	0	0	0.33	0.95	35
**31**	EAP60-1	3.37	2.28	2.89	43.61	37.67	0	10.18	0	0	0	0.48	1.49	35
**32**	EAP80-2	1.22	0	6.73	21.64	55.56	10.39	4.46	0	0	0	0.14	1.84	35
**33**	PS-2	4.17	17.33	18.65	35.14	19.11	0	5.59	0	0	0	0	0.89	75
**34**	EUPS-2	8.83	15.77	12.39	43.94	11.15	0	7.92	0	0	0	0	1.36	75
**35**	CMP-1	4.2	0	0	95.8	0	0	0	0	0	0	0	0.65	36
**36**	EPS-1	0	0	10.6	84	5.4	0	0	0	0	0	19.86	4.84	76
**37**	EPS-2	0	0	32.7	57.3	10.9	0	0	0	0	0	20.3	2.69	76
**38**	IPS-1	0	0	59	8.5	32.6	0	0	0	0	0	33.97	1.32	76
**39**	IPS-2	0	0	42.2	19.8	38	0	0	0	0	0	20.38	1.58	76
**40**	IPS-3	0	0	27.2	72.8	0	0	0	0	0	0	1.9	1.91	76
**41**	PPM	0	0	69.1	7.8	23.1	0	0	0	0	0	0	1.99	64
**42**	GPA1	0.4	21.2	10.6	13.8	27.5	2.2	0	14.8	9.5	23.04	3.75	0.22	37
**43**	GPA2	0.8	15.6	8.2	18	21.4	1.6	1.6	18	14.8	32.79	4.38	0.21	37
**44**	GPA3	3.8	7.5	6.3	34.3	16.3	1.3	3.1	24.3	3.1	27.01	5.53	0.2	37
**45**	RCP-II	9.8	21.3	0	7.9	33.8	0	9.3	0	17.9	23.6	0	0.96	77
**46**	AAP-2A	8	25.7	0	49.3	17	0	0	0	0	0	0	0.022	65
**47**	TPC	0	12.7	0	11.2	5.4	0	33.8	27.1	0	0	0	0.101	78
**48**	TPC-1	0	21.2	16	26.3	6.4	0	17.3	0	0	30	2.8	0.184	79
**49**	TPC-2	0	26.4	13.9	37.5	0	0	0	0	0	47.6	3.8	0.158	79
**50**	TPC-3	0	37.2	0	14.9	8.3	0	23.1	0	0	51.8	4	0.093	79
**51**	GO-2	24.2	0	0	25.8	0	0	0	50	0	0	0	1.13	80
**52**	GO-3	24.5	0	0	14.1	0	0	0	61.4	0	0	0	0.93	80
**53**	GO-4	22.4	0	0	0.7	0	0	0	76.9	0	0	0	0.7	80
**54**	RNLP I	10.1	51.7	3.5	22.3	8.8	0	3.6	0	0	6.71	0	1.74	66
**55**	PSCK2-2	4	12.4	43.3	0	0	36.4	0	0	4	24.7	0	1.5	81
**56**	PSCK2-3	5	11.3	45.7	2.5	0	35.5	0	0	0	6.64	0	4.8	81
**57**	APs-1-1	1.4	8.1	0	68.2	0	0	22.3	0	0	0	3.1	0.2092	40
**58**	APs-2-1	4.6	8	0	32.3	24.2	0	21.1	0	9.8	0	1.9	0.1967	40
**59**	APs-3-1	1.5	2.8	0	35.1	34	0	16.7	2	7.9	0	1.3	0.1715	40
**60**	WSEPS	0	14.5	0	31.9	40.6	0	0	13	0	0	0	0.07	82
**61**	CT-EPS	11.4	7.4	19	40	13.5	0	8.7	0	0	0	14.87	1.62	42
**62**	PSS-EPS	8.2	7.7	24	35.3	15.4	0	9.4	0	0	0	20.19	1.119	42
**63**	PSS-DEPS	3.3	5.6	25.5	31.5	29.8	0	4.3	0	0	0	26.47	3.522	42
**64**	CT-IPS	2.1	6.2	18	59.7	9	0	5	0	0	0	25.06	8.828	42
**65**	PSS-IPS	1.7	6.9	8.6	73.1	5	0	4.7	0	0	0	10.82	0.779	42
**66**	PS2	10.96	5.81	36.16	26.92	14.55	4.52	1.04	0	0	0	0	0.98	44
**67**	PS3	48.55	10.73	7.35	11.41	13.85	4.62	3.45	0	0	0	0	0.66	44

^a^name from reference

^b^rhamnose

^c^arabinose

^d^mannose

^e^glucose

^f^ galactose

^g^fucose

^h^xylose

^i^glucuronic acid

^j^galacturonic acid

^k^uronic acid

^l^protein content

We selected five relevant descriptors in MLR model, and a stable model EC_50_ = 0.12PC+0.083Fuc+0.013Rha-0.02UA+0.372 (R = 0.664, RMSE = 1.149, F = 8.268, p<5.17E^-5^) was given. According to the model, PC, Fuc, Rha and UA had significant correlation with EC_50_ of the hydroxyl radicals scavenging activity, and the relevant correlation coefficient was shown in Table 6.

**Table 6:** Correlation matrix showing inter-correlation among various parameters and EC_50_ of the hydroxyl radicals scavenging activity.

	EC_50_	PC	Fuc	Rha	UA
EC_50_	1.000000				
PC^a^	0.515359	1.000000			
Fuc^b^	0.270504	-0.134435	1.000000		
Rha^c^	-0.093930	-0.125825	-0.017084	1.000000	
UA^d^	-0.126494	-0.028296	0.167403	-0.180576	1.000000

^a^protein content

^b^fucose

^c^rhamnose

^d^uronic acid

The statistical parameters of MLR, ANN and SVM models for the train set and the test set were shown in Table 7. According to a lower RMSE and a higher R value, the results indicated that nonlinear model ANN was better than models obtained from MLR and SVM for the prediction of hydroxyl radicals scavenging activity of polysaccharides.

**Table 7:** Comparison of MLR, ANN and SVM models for the hydroxyl radicals scavenging activity of polysaccharides.

Method	Parameters	Training set	Test set
MLR	R	0.664	0.523
	RMSE	1.149	1.117
ANN	R	0.944	0.857
	RMSE	0.119	0.257
SVM	R	0.836	0.767
	RMSE	0.751	0.645

### Sensitivity analysis from ANN

According to two ANN models, the results of sensitivity analysis were shown in Table 8. The higher sensitivity coefficient indicated that this descriptor had the more influence upon the antioxidant activity of polysaccharides. The results indicated that Ara and GalA had a great effect on DPPH-scavenging activity, and PC, UA and GalA had a great effect on hydroxyl radicals scavenging activity of polysaccharides, which was consistent with the results from MLR.

**Table 8:** Sensitivity analysis from ANN models.

Sensitivity coefficients	Composition										
	Ara^a^	GalA^b^	PC^c^	GlcA^d^	UA^e^	Glc^f^	Xyl^g^	Man^h^	Gal^i^	Fuc^j^	Rha^k^
DPPH-scavenging activity	6.48	3.4	3.25	2.98	2.61	1.52	1.29	1.23	1.11	1.11	0.97
Hydroxyl radicals scavenging activity	1.85	4.76	7.37	1.21	3.78	3.24	1.08	1.49	2.9	3.44	1.35

^a^arabinose

^b^galacturonic acid

^c^protein content

^d^glucuronic acid

^e^uronic acid

^f^glucose

^g^xylose

^h^mannose

^i^galactose

^j^fucose

^k^rhamnose

## Conclusions

To establish quantitative structure-activity relationship (QSAR) models for antioxidant activity of polysaccharides, MLR, SVM and ANN methods were used, and polysaccharide properties (UA, PC, monosaccharide compositions, MW) as descriptors were selected. MLR models for predicting EC_50_ of DPPH-scavenging activity and hydroxyl radicals scavenging activity of polysaccharides consisted of four major descriptors, and the models were EC_50_ = 0.033Ara- 0.041GalA- 0.03GlcA- 0.025PC +0.484 and EC_50_ = 0.12PC +0.083Fuc +0.013Rha -0.02UA+0.372, respectively. A comparison of results from models indicated that the ANN model with R = 0.96 and RMSE = 0.018 predicted more accurately the DPPH-scavenging activity of polysaccharides than SVM and MLR models. ANN model (R = 0.933, RMSE = 0.055) was also the best one for predicting the hydroxyl radicals scavenging activity of polysaccharides. According to MLR and ANN models, Ara and GalA were most critical in determining the DPPH-scavenging activity of polysaccharides, and PC, UA and GalA had a great effect on hydroxyl radicals scavenging activity of polysaccharides. The polysaccharide of MW 4000–100000 usually owned higher DPPH-scavenging activity, but the antioxidant activity could simultaneously be affected by other polysaccharide properties. These results may provide some new insights in the complex study of polysaccharide structure and bioactivities, and we can simply predict the antioxidant activity of polysaccharide by using the established models after determining the monosaccharide composition ratios and MW.

It is worth noting that the highly GalA-containing polysaccharide could exhibit significantly antioxidant activity, which might be because they owned the functional group–COOH. It has been reported that the functional groups such as–COOH, CH_3_CO–and–SH were generally recognized as good electron or hydrogen donors that might be related to the antioxidant activity of polysaccharides [5]. The antioxidant activity of polysaccharide was also found to correlate to complex structure such as glycosidic linkages, branch ratios, and microstructure etc, polysaccharide properties is not enough for fine detailed structure of polysaccharide, and the research on more precise structure-function relationships remained to be explored.
